# Wound healing potential of human umbilical cord mesenchymal stem cell conditioned medium: An *in vitro* and *in vivo* study in diabetes-induced rats

**DOI:** 10.14202/vetworld.2021.2109-2117

**Published:** 2021-08-17

**Authors:** Siufui Hendrawan, Yuyus Kusnadi, Christine Ayu Lagonda, Dilafitria Fauza, Jennifer Lheman, Erwin Budi, Brian Saputra Manurung, Hans Ulrich Baer, Sukmawati Tansil Tan

**Affiliations:** 1 Department of Biochemistry and Molecular Biology, Faculty of Medicine, Tarumanagara University, 11440, Jakarta, Indonesia; 2 Tarumanagara Human Cell Technology Laboratory, Tarumanagara University, 11440, Jakarta, Indonesia; 3 Stem Cell Division, Stem Cell and Cancer Institute, PT. Kalbe Farma, Tbk., 10510, Jakarta, Indonesia; 4 Baermed, Centre of Abdominal Surgery, Hirslanden Clinic, 2501, Zürich, Switzerland; 5 Department of Visceral and Transplantation Surgery, University of Bern, 3012, Bern, Switzerland; 6 Department of Dermatovenereology, Faculty of Medicine, Tarumanagara University, 11440, Jakarta, Indonesia

**Keywords:** conditioned medium, diabetic induced rat, human umbilical cord mesenchymal stem cells, wound healing

## Abstract

**Background and Aim:**

Human umbilical cord mesenchymal stem cells (hUC-MSCs) and its conditioned medium (CM) promote wound healing. This study investigated the wound healing potential of hUC-MSC CM *in vitro* and *in vivo* using diabetic animal models.

**Materials and Methods:**

The CM from hUC-MSC CM prepared under hypoxic conditions (hypoxic hUC-MSC) was evaluated for stimulating rat fibroblast growth, collagen production (*in vitro*), and wound healing in animal models (*in vivo*). An excision wound on the dorsal side of the diabetes-induced rats was established, and the rats were randomly divided into non-treatment, antibiotic, and hypoxic hUC-MSC CM groups. The cell number of fibroblasts and collagen secretion was evaluated and compared among the groups in an *in vitro* study. By contrast, wound size reduction, width of re-epithelialization, and the collagen formation area were assessed and compared among the groups in an *in vivo* study.

**Results:**

CM under hypoxic conditions contained a higher concentration of wound healing-related growth factors. Hypoxic hUC-MSC CM could facilitate fibroblast cell growth and collagen synthesis, although not significant compared with the control group. Re-epithelialization and collagen production were higher in the hUC-MSC CM group than in the antibiotic and non-treatment groups.

**Conclusion:**

Hypoxic hUC-MSC CM possessed more positive effects on the wound healing process based on re-epithelialization and collagen formation than antibiotic treatment did.

## Introduction

Chronic wounds have become a global public health challenge. Unlike acute wounds, which heal without significant interventions, chronic wounds give major challenges to patients and doctors. There are varying etiologies of chronic wounds, and one of them is diabetes [1]. The prevalence of patients with diabetes worldwide is predicted to rise by 5.4% (300 million) by 2025 [2]. Ulcer in patients with diabetes is common, and diabetic foot ulcer in patients is a severe and complex case [3]. Diabetic foot ulcers significantly influence the quality of life of patients, including limited and reduced mobility, diminished income, loss of job, and spending more to visit a physician or clinic for care [4]. Diabetes influences many aspects of life in patients, such as health, social, and economy. In the economic aspect, the cost of managing patients with diabetes with a lower extremity ulcer has a more economic burden than diabetic patients without ulcers [5]. Alternative solutions are needed to overcome the severe impacts that arise from diabetes, especially diabetic-related wounds. A diabetic wound is associated with decreased peripheral blood flow. Impaired angiogenesis and neovascularization result in insufficient oxygen and nutrient supply for the cells, leading to further impaired healing. Healing deficiency of diabetic wounds can be attributed to other factors, including decreased production of growth factors and reduced revascularization. A diabetic wound is challenging to treat and requires comprehensive procedures. The standard management of diabetic wounds includes surgical debridement, vascular assessment, infection treatment, and glycemic control. Even with holistic approaches, adjuvant therapies are still needed to improve healing times [6,7].

Wound repair processes do not work smoothly in chronic wounds, like diabetic ulcers, because of several factors such as microbial infection [8], biofilm formation [9], and excessive inflammatory phase [10]. Recently, stem cells have been proven to have therapeutic potential. Mesenchymal stem cells (MSCs) are multipotent nonhematopoietic progenitor cells that show great promise for tissue regeneration. MSCs isolated from the umbilical cord and its conditioned medium (CM) can be easily obtained and refined compared to stem cells from other sources [11,12]. MSCs play a key role in three stages of the wound healing process and promote fibroblast migration during re-epithelialization [13]. The main concern in wound repair is inflammation. This concern can be solved by MSC characteristics that could reduce wound inflammation by repressing the proliferation of host T-cells [14,15]. The use of human umbilical cord-derived MSCs for tissue repair provides some advantages compared with other sources of MSCs, including cost-effectiveness, easy isolation process, low invasiveness, and low immunogenicity [16]. In addition, human umbilical cord-derived MSCs have other main characteristics, such as high proliferative ability and oxidative stress protein expression [17]. The previous studies demonstrated that umbilical cord MSCs have superior wound-healing capability either in an *in vitro* study [18] or in diabetic rats (*in vivo* study) [19]. Human umbilical cord MSCs (hUC-MSCs) were superior to fibroblasts in stimulating diabetic wound healing, marked by higher cell proliferation, collagen synthesis, and glycosaminoglycan level [20]. Another study also reported the efficacy of hUC-MSCs in stimulating cutaneous healing in burned rats [21]. Both UC-MSC and their CM were beneficial to diabetic wound healing, and in the context of wound healing application, CM is better than the cell itself [12]_._

As reported in a previous study, stem cells have essential roles in tissue regeneration conferred by its secreted paracrine factors, known as the secretome [22]. The secretome is defined as various molecules secreted from stem cells, such as cytokines, chemokines, and growth factors [23]. These molecules are secreted into the cell culture medium called CM [22]. Dressing and topical products, including topical antiseptic and antimicrobial application, are adjuvant therapies that are commonly used for diabetic foot ulcer care [3]. However, dressing and topical products might have limitations in curing diabetic foot ulcers. Furthermore, no studies have reported the efficacy comparison of hUC-MSC CM with antibiotics in wound repair.

Therefore, this study was conducted to evaluate the effect of hUC-MSC CM that was prepared under hypoxic conditions in stimulating rat fibroblast growth and collagen production (*in vitro* study) and to evaluate the treatment effect of hypoxic hUC-MSC CM on wound healing in diabetes-induced rats compared to antibiotic treatment (*in vivo* study).

## Materials and Methods

### Ethical approval

Animal experiments in this study were approved by Institutional Animal Care and Use Committees (IACUC) (001.KEPH/UPPM/FK/IV/2019) of the Faculty of Medicine, Tarumanagara University. Surgical procedures for obtaining the fibroblast were conducted according to protocols approved by the Tarumanagara University IACUC, with IACUC approval number 003.KEPH/UPPM/FK/VI/2019.

### Study period and location

The study was conducted from May 2019 to June 2020 at Tarumanagara Human Cell Technology Laboratory, Faculty of Medicine, Tarumanagara University, Indonesia.

### hUC-MSC CM preparation

#### Human umbilical cord isolation

Fresh umbilical cords were collected after obtaining parental consent. The umbilical cords were washed with phosphate-buffered saline containing antibiotic–antimycotic (Ab–Am) solution before isolation. Veins and arteries were carefully removed from the umbilical cord. The Wharton jelly of the umbilical cord was then cut into small pieces and carefully placed on a 100-mm culture dish, which was filled with cell culture medium consisting of alpha minimum essential medium (a MEM; Gibco by Life Technologies, Grand Island, NY, USA) supplemented with 10% fetal bovine serum (FBS; Gibco by Life Technologies) and 1% Ab–Am (Gibco by Life Technologies). For 21 d, MSC was migrated out from the umbilical cord tissue and harvested for further expansion.

#### Identification of MSCs

Immunophenotyping assay was conducted to ensure that the characteristic of the MSCs was in accordance with theInternational Society for Cell and Gene Therapy (ISCT) guidelines [24]. The surface markers CD73, CD105, and CD90, conjugated with phycoerythrin (R and D Systems), were used to stain MSCs. The assay used flow cytometry (FACS Calibur, BD, USA), and the analysis used CellQuest Pro software. The minimum acceptance criteria were 95% for all positive markers [24].

#### hUC-MSC CM production

MSCs isolated from the umbilical cord were cultured until passage 6 (P6) in a T175 flask (Corning). The culture medium was deprived and replaced with basal medium with no supplement addition after reaching 70%–80% confluence and then incubated at 37°C in a 5% CO_2_ incubator with two oxygen conditions: normoxic (21% O_2_) and hypoxic (5% O_2_). After 72 h of incubation, the hUC-MSC CM was collected and stored in a deep freezer (−80°C) for long-term storage. hUC-MSCs were isolated and cultured at the Stem Cell and Cancer Institute Laboratory, Jakarta, Indonesia.

#### Quantification of basic fibroblast growth factor (bFGF), vascular endothelial growth factor (VEGF), and pro-collagen 1 as wound healing-related paracrine factors

Enzyme-linked immunosorbent assay (ELISA) was performed to quantify paracrine factors secreted by MSCs related to wound healing, such as bFGF, VEGF, and pro-collagen 1 (R and D Systems). The procedure was performed according to the manufacturer’s instructions, and the analysis was performed using four-parameter logistic software.

### *In vitro* study

#### Rat fibroblast cell growth

Primary rat dermal fibroblasts were used for the *in vitro* study. MSCs (passage 5–6) cultured under hypoxic conditions for 72 h were used to produce the hUC-MSC CM. In addition, rat fibroblasts were obtained from donor rats. One male 12-week-old *Sprague–Dawley* rat (National Agency of Drug and Food Control, Jakarta) was used as a fibroblast donor. Fibroblasts were isolated from the rat’s skin as described previously [25]. Fibroblasts at a density of 5×10^5^ cells/well were seeded into 24-well plates (n=3) and then cultured in Dulbecco’s modified Eagle medium (DMEM; Gibco by Life Technologies) supplemented with 10% (v/v) FBS and 1 mL of penicillin/streptomycin (1×). hUC-MSC CM was added to the media culture with two concentrations, namely, 0.5% and 1%. As a control, the same number of fibroblasts was seeded into wells and cultivated in the media without adding the hUC-MSC CM. Cells were incubated at 37°C in 5% CO_2_ for 24 h. The cell number was analyzed using the Cell Counting Kit-8 (CCK-8) viability assay (Sigma Aldrich, USA) and measured in a Multiskan reader at a wavelength (l) of 450 nm (Multiskan Ex, Thermo Scientific, USA).

### Analysis of collagen production on rat fibroblasts

Collagen production by fibroblasts was measured using rat type I collagen ELISA kit (Elabscience Biotechnology Inc., China). Fibroblasts (5×10^5^ cells/well) were seeded into 24-well plates, cultivated in Dulbecco’s modified eagle medium (Gibco) supplemented with 10% (v/v) FBS and 1 mL of penicillin/streptomycin (1×), and incubated at 37°C in 5% CO_2_ for 24 h. The procedure was performed according to the manufacturer’s instructions. The rat fibroblast collagen concentration was obtained using an ELISA reader at a wavelength (λ) of 450 nm.

### *In vivo* study

#### Rat skin-wound healing model and induction of diabetes in rats

Nine adult male, 14-week-old Sprague–Dawley rats weighing 150-180 g were obtained from the National Agency of Drug and Food Control, Jakarta, Indonesia. Before the experimental procedures, the animals were acclimatized to the laboratory conditions. They were allowed free access to standard laboratory food and water and housed individually in cages in a controlled environment (23°C±3°C, 30-70% humidity, and a 12:12 h light: dark cycle). For diabetes induction, the rats were rendered diabetic 1 week before treatment by a single-dose intraperitoneal injection of streptozotocin (STZ) (50 mg/kg body weight; Selleck Chemicals). Blood glucose was checked 48 h post-injection, and blood samples were obtained from the tail vein of the animals for glucose assay. The rats were considered diabetic if three days after the STZ injection, the non-fasting blood glucose was more than 200 mg/dL and presented with at least 3 days of persistent hyperglycemia.

The diabetes-induced rats were randomized and divided into the following three treatment groups (n=3): Which were non-treatment (without any medication), antibiotics (topical medication with bactoderm mupirocin ointment 2%, Ikapharmindo Putramas, Indonesia), and hUC-MSC CM groups (intradermal injection with 0.5 mL of undiluted CM on a peripheral wound). For the experimental procedure, the rats were weighed and anesthetized by intraperitoneal administration of ketamine 10% INJ (Kepro B.V, Holland; 40-80 mg/kg body weight) and x ylazine2% (Interchemie werken “De Adelaar” B.V. Metaalweg 8 Venray, Holland; 5-10 mg/kg body weight). Then, the hair on the backside was shaved and disinfected. The skin on the dorsal area of the rats was punctured using an 8-mm skin punch, with each rat having two ulcer wounds and receiving different treatments. After the procedure, wounds were closed with Tegaderm (3M Nexcare) and fixed with a suture at 3-4 points. Hypafix (Essity, Healthcare 21 Group, Ref. 71442-03) was added to secure the Tegaderm. Three wounds per treatment were analyzed in this study. The wound observations, photos, and measurements were conducted at three endpoints: 0, 7, and 14 d post-skin puncture. The percentage of wound closure was calculated using the equation: percentage of wound closure (%) = (A_0_-A_t_)/A_0_×100%, where A_0_ is the wound area at Day 0 and A_t_ is the wound area at 7 or 14 d post-skin puncture.

### Histopathological analysis

All *Sprague-Dawley* rats were euthanized at 14 d post-skin puncture. Skin specimens from the wound site were dissected and collected. Specimens were fixed in 10% formalin and sent to the Pathology and Anatomy Laboratory, Primate Research Center, Bogor Agricultural University, for further examinations, including re-epithelialization with hematoxylin and eosin (HE) staining and collagen formation area measurement with Masson’s trichrome staining. HE staining and MT staining were performed according to Sheehan and Hrapchak [26]. Tissue sections were observed and captured using a Nikon Eclipse 80i microscope to evaluate re-epithelialization and collagen-deposited areas. Furthermore, the width of re-epithelialization and collagen-deposited areas was analyzed using the ImageJ software.

### Statistical analysis

All data were presented as mean±SD. The differences between the two independent groups were analyzed by Mann–Whitney and Kruskal–Wallis tests to analyze the differences among the three independent groups. Statistical analysis was conducted using SPSS v.23, with a 95% confidence interval. Values were considered statistically significant at p<0.05.

## Results

### hUC-MSC characteristics

**Figure-1:**
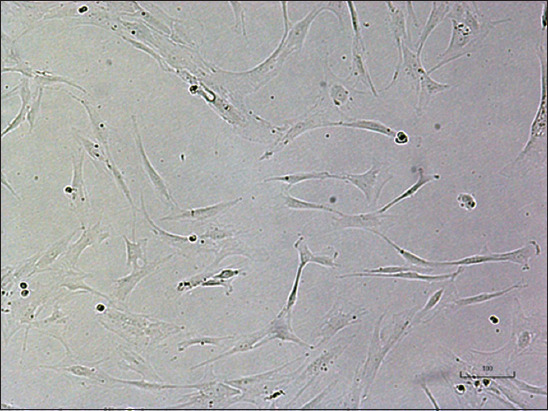
Microscopic Appearance of human umbilical cord mesenchymal stem cells (passage 5) with magnification 400×. Scale bar: 100 μm.

The human MSCs obtained from umbilical cord primary isolation were 1×10^5^ cells and expanded from passage 0-6. Culture expansion used a cell density of 7×10^3^ cells/cm^2^ as the seeding concentration standard. hUC-MSC morphology was like a spindle-shaped fibroblast (Figure-1), and the size of the MSC was approximately 120 μm. There was no significant change shown by MSCs based on their morphology throughout culturing.

MSCs expressed markers CD73, CD90, and CD105 and did not express hematopoietic markers such as CD34, CD45, CD14, and CD19. The flow cytometry results confirmed that the cells used were MSCs, with values of all positive markers above 95% and negative markers under 2% (Table-1). The surface epitopes are considered MSC-specific markers according to the ISCT [24].

**Table-1:** Marker analysis of obtained mesenchymal stem cells (MSCs) from human umbilical cord.

Markers	Population percentage
CD105	99.47
CD73	99.26
CD90	99.52
CD14	0
CD45	0.26
CD19	1.47

### Pro-collagen, VEGF, and bFGF Content in hUC-MSC CM

**Figure-2:**
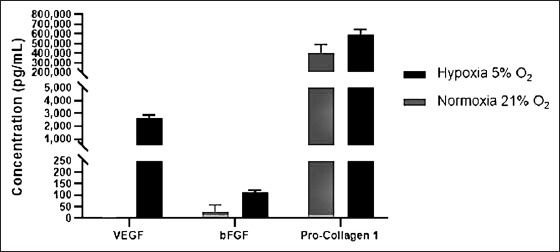
Vascular endothelial growth factor, basic fibroblast growth factor, and pro-collagen 1 amount produced from human umbilical cord mesenchymal stem cells cultured in normoxic and hypoxic conditions. Data show mean±SD.

We used passage six cells for CM production because of their defined characters of MSC. The MSC cells were cultured with MEM-Alpha without an additional growth supplement after reaching 70-80% confluence. The MSC cells were further incubated for 72 h to allow the secretion of the secretome. To investigate the favorable condition of secretome secretion, we placed the MSCs under two conditions (normoxic [21% O_2_] and hypoxic [5% O_2_]). The CM was stored in a deep freezer to maintain the stability of its protein content for long-term storage. On the basis of the ELISA results of CM from normoxic and hypoxic hUC-MSC, it can be concluded that there were concentration differences for all types of secreted protein between the two conditions (Figure-2). In CM from hUC-MSC hypoxic conditions, VEGF, bFGF, and pro-collagen 1 concentrations were higher than those in normoxic conditions. Furthermore, in CM from the hUC-MSC normoxic conditions, VEGF was not secreted, whereas, in CM from the hypoxic condition, it was secreted more than 2 ng/mL. In line with these results, bFGF and pro-collagen 1 secretions were highly secreted under hypoxic conditions. These results indicate that the incubation of hUC-MSCs under hypoxic conditions was stimulated to secrete more VEGF, bFGF, and pro-collagen 1. CM from hypoxic hUC-MSC was used in further *in vitro* and *in vivo* trials based on these results.

### Cell growth of rat fibroblast

**Figure-3:**
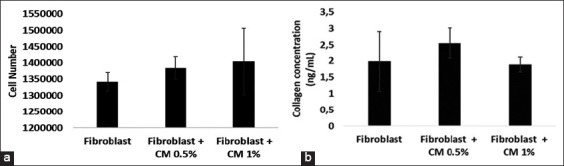
(a) Cell number of fibroblasts treated with two different concentrations of conditioned medium (CM) and (b) collagen concentration secreted from fibroblast post-treated with human umbilical cord mesenchymal stem cells CM 0.5% and 1% concentrations (n=3). Data show mean±SD.

Fibroblasts were treated with two concentrations of hypoxic hUC-MSC CM (0.5% and 1%) to observe cell growth and function. The cell number of fibroblasts treated with CM 1% at 24 h after cell seeding was higher than the cell numbers of those treated with CM 0.5% and untreated. However, the mean cell number of the three groups did not differ significantly (control group: 1.34×10^6^±2.83×10^4^; CM 0.5% group: 1.38×10^6^±3.38×10^4^; CM 1% group: 1.40×10^6^±1.02×10^5^) (Figure-3a). Although the results showed that a higher concentration of hypoxic hUC-MSC CM might better facilitate fibroblast growth, statistically, there was no significant difference in the cell number of the fibroblast (Figure-3a). These results showed that adding a certain concentration of CM did not significantly affect fibroblast cell growth.

### Collagen secretion

To examine the effect of adding hypoxic hUC-MSC CM against fibroblast function in producing collagen, we also used two concentrations of CM. Hypoxic hUC-MSC CM (both 0.5% and 1%) showed improved collagen secretion than the control group did. However, the mean collagen concentration was not significantly different between groups (control group: 1.99±0.91 ng/mL; CM 0.5% group: 2.55±0.47 ng/mL; CM 1% group: 1.89±0.23 ng/mL) (Figure-3b). Our study may indicate that collagen production did not strongly depend on CM, and the lower or higher concentration of CM did not significantly increase collagen production.

### Wound healing observation in diabetic ulcer

**Figure-4:**
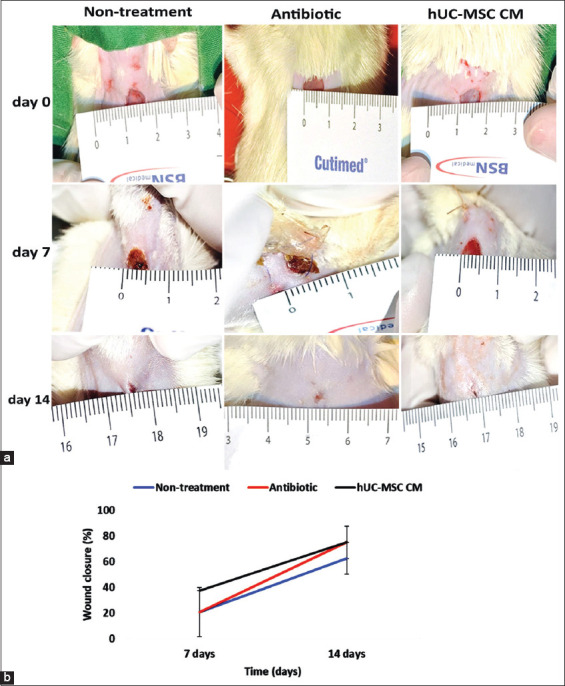
(a) Wound closure measurement on diabetic induced rats (non-treatment, antibiotic and human umbilical cord mesenchymal stem cells conditioned medium groups) at three endpoints (0-, 7-, and 14-days). (b) Percentage of wound closure comparison between groups at 7- and 14-days post-skin puncture.

**Figure-5:**
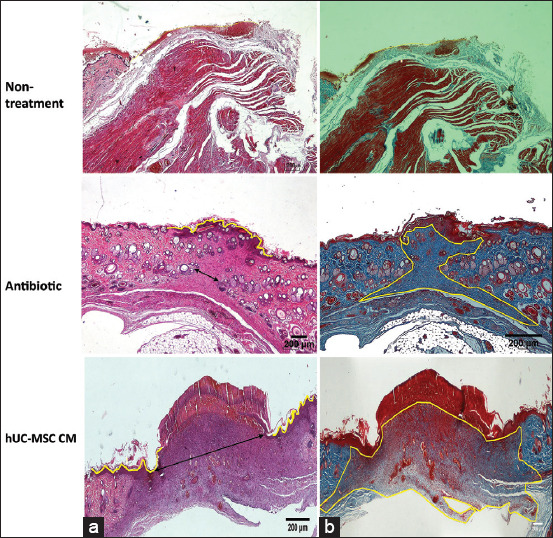
Histopathological analysis of (a) re-epithelialization using Hematoxylin and Eosin staining and (b) collagen formation using Masson’s trichrome staining on the wound site of diabetic induced rats. Re-epithelialization and collagen formation areas are indicated with yellow lines. Scale bar 200 µm in all panels. The double-headed arrows point the edge of the scar.

The wound healing process was observed by re-epithelialization and newly synthesized collagen. In addition, different responses were observed between the non-treatment, antibiotic, and hypoxic hUC-MSC CM groups. Our preliminary experiment showed that CM obtained from hypoxic hUC-MSC showed a superior effect in wound healing compared with that from normoxic hUC-MSC. On the basis of these findings, we treated the wound with CM of MSCs cultivated under hypoxic conditions. As hypothesized, the hypoxic hUC-MSC CM demonstrated wound healing potential in diabetic animal models, both in macroscopic (Figure-4a) and microscopic observations (Figure-5).

At the macroscopic observation, the hypoxic hUC-MSC CM group demonstrated faster wound closure post-skin puncture. Figure-4b showed the highest wound closure percentage on wounds treated with hypoxic hUC-MSC CM (37.5%±0%) after 7 d post-skin puncture compared with the antibiotic (20.83%±19.09%) and non-treatment groups (20.83%±19.09%). The hypoxic hUC-MSC CM treated wound showed better and faster wound closure than that treated with an antibiotic and untreated wound at 7 d post-skin puncture. The hypoxic hUC-MSC CM group had the same wound closure percentage (75%±12.5%) as that with the antibiotic group (75%±0%) and had a higher wound closure percentage than that with the non-treatment group (62.5%±12.5%) at 14 d post-skin puncture (Figure-4a). The wound closure percentage in wounds treated with hypoxic hUC-MSC CM was the same as that of the antibiotic group at 14 d, but still higher than that of the non-treatment group (Figure-4b).

**Figure-6:**
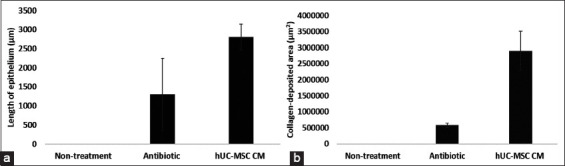
(a) Length of epithelium and (b) width of collagen-deposited area comparison between non-treatment, antibiotic, and human umbilical cord mesenchymal stem cells conditioned medium group in diabetic induced rats at 14 days post skin punctures.

The hypoxic hUC-MSC CM group exhibited the largest re-epithelialization area for microscopic observation compared with the antibiotic group (Figure-6a). Moreover, there was no re-epithelialization area found in the non-treatment group. In addition, the epithelium length (2806±338 μm) of the hypoxic UC-MSC CM group was 2.15-fold longer than the epithelium length of the antibiotic group (1303±940 μm) (Figure-6a). These results were also supported by the width of the collagen formation area data (Figure-6b). Correspondingly, the area of the newly generated collagen tissue in the hUC-MSC CM group (29.03×10^4^±60.99×10^4^ μm^2^) was five-fold wider than that in the antibiotic group (57.93×10^4^±64.77×10^4^ μm^2^) (Figure-6b). Altogether, these findings demonstrated that hypoxic hUC-MSC CM treatment showed a distinct effect to facilitate wound repair in our diabetic wound model.

## Discussion

This study analyzed the efficacy of hypoxic hUC-MSC CM on wound healing of diabetic rats. We also performed antibiotic treatment for comparison. First, we confirmed that our cells were MSCs based on morphology observation and supported by marker analysis results (Figures-1 and 2). To investigate the suitable conditions to produce CM, we observed two MSC-cultivated conditions (normoxic and hypoxic conditions). Interestingly, the production levels of VEGF, bFGF, and pro-collagen 1 were different. The hypoxic condition increased the production of the CM by MSCs. It has been reported that the mRNA level and protein expression of VEGF-A were higher in MSCs prepared under hypoxic conditions compared with normoxic conditions [27]. Another study by Page *et al*. [28] also reported that gene expression and paracrine secretion are strongly affected by serum and oxygen concentration. Our study observed that there was no VEGF production by the MSCs under normoxic conditions. In contrast to our findings, Chen *et al*. [29] investigated that VEGF-A was still produced at a lower concentration under normoxic conditions by bone marrow MSCs than under hypoxic conditions. However, our result in bFGF was supported by their findings. Meanwhile, we found that pro-collagen 1 was secreted in these two setup conditions, and their concentrations were slightly different. These data suggested that hypoxic conditions were preferable to stimulate the secretion of secretome/paracrine. Similar to our finding, Hsiao *et al*. [30] also demonstrated that hypoxic conditions favored VEGF-A and ANG production as angiogenic factors. Likewise, a previous study reported that VEGF and bFGF induce angiogenesis in wounded skin [31]. VEGF acts on wound repair through angiogenesis, collagen synthesis, and epithelialization [32].

To explore the effect of hypoxic hUC-MSC CM on fibroblast cell growth and collagen secretion *in vitro*, we treated fibroblast with hypoxic hUC-MSC CM with two concentrations (0.5% and 1%). Our findings showed that hypoxic hUC-MSC CM increased the number of fibroblasts, particularly in the group treated with a higher concentration (1%). Thus, it suggested that hypoxic hUC-MSC CM addition exerted a positive effect on fibroblast proliferation. Kim *et al*. [33] reported a similar result, and they investigated the highest growth of HDF, cell concentration, and protein level observed in the USC-CM group. They also evaluated the activity of USC-MSC to induce migration of HDF compared with HDF and another MSC-CM group. Other studies also proved that the application of MSC-CM facilitated higher fibroblast proliferation than those in the control group [34].

Our findings showed that adding hypoxic hUC-MSC CM enhances collagen secretion, especially in fibroblasts treated with hypoxic hUC-MSC CM 0.5%. It may indicate that the collagen production did not strongly depend on the concentration of used hypoxic hUC-MSC CM. The previous studies by Kim *et al*. [33] demonstrated that the production of ECM (collagen, elastin, and fibronectin) was also triggered by USC-CM. Villanueva *et al*. [35] examined a lower collagen production from lung fibroblasts found in the control group (without CM addition) compared with adding endothelial cell-derived CM (1:10) at a 6-h incubation. At 24-h incubation, treatment with an increasing concentration of CM elevated the amount of collagen synthesized. As one of the extracellular matrix compositions in the skin, collagen plays a key role in growth and determines skin’s elasticity [36].

To investigate the therapeutic effect of hypoxic hUC-MSC CM *in vivo*, we provided an antibiotic group for comparison and the non-treatment as a control group. We chose topical antibiotic treatment because of its ability to inhibit or kill microbes on an open wound. Our results showed that the hypoxic hUC-MSC CM group had a beneficial effect on the wound healing process, which was revealed by macroscopic and microscopic observation. An expected outcome was a reduction of the wound surface area. Although not statistically significant, the hUC-MSC CM group demonstrated faster wound closure since observation at 7 d post-skin puncture. Non-significant results might be caused by the small sample size and the length of the observation time. The created wound site was reduced at 14 d post-wounding. The hypoxic hUC-MSC CM group had the same wound closure percentage as that of the antibiotic group and had a higher wound closure percentage than that of the non-treatment group at 14 d post-skin puncture. A study by Han *et al*. [19] showed a wound healing process that was significantly lower in diabetic rats than in normal rats at days 7 and 14. This phenomenon in their study showed that blockade of the Wnt signaling pathway slowed the healing of skin wounds in diabetic rats. A study by Assi *et al*. [37] in mice showed that wounds treated with topical scaffolds containing MSCs increased proliferation without increasing apoptosis and VEGF-positive cells. Another study by Zhou *et al*. [38] demonstrated that hUC-MSC CM/hydrogel promoted wound closure and constricted fibrotic and hypertrophic scar tissue formation.

The efficacy of antibiotics to facilitate wound healing was not as high as that triggered by hUC-MSC CM application but was better than the non-treatment group. Reports from Padeta *et al*. [34] demonstrated a similar finding to our results. They found that the MSC CM showed a prominent effect in promoting wound healing than bioplacenton application as their control group prepared from bovine placenta extract 10%, neomycin sulfate 0.5%, and gel base. Thus, besides the self-renewal capacity, human MSCs and their secretome may have antimicrobial activity. Our hypothesis was supported by Krasnodembskaya *et al*. [39], who observed the antimicrobial efficacy of human MSCs and their CM. This antimicrobial activity was exerted by the antimicrobial peptide LL-37 production. These results suggest that using hUC-MSC CM had a superior effect compared with antibiotics application alone. These benefits might trigger the efficacy of hypoxic hUC-MSC CM.

Wound healing can be characterized by re-epithelialization [40]. As a further investigation, we performed the histopathological analysis of our specimen to observe the effect of given treatments on wound repair. We observed and measured the newly formed epithelial tissue and collagen-deposited areas in hypoxic hUC-MSC CM and the topical antibiotic group. It indicated that the treated skin was at the proliferation phase. Furthermore, the proliferation phase in wound repair is marked by four processes, including re-epithelialization, angiogenesis, collagen synthesis, and extracellular formation [41,42]. In re-epithelialization, the new epithelium formed by proliferation, migration, and keratinocyte differentiation also protects beneath the epidermal layer [43].

Histopathological analysis of a wound site showed an increase in re-epithelialization area in the hypoxic hUC-MSC CM group compared with the non-treatment and antibiotic groups at 14 d post-skin puncture. A similar study reported by Shrestha *et al*. [12] showed enhanced re-epithelialization after hUC-MSC CM application on the wound at 14 d post-treatment [9]. Consistent with the re-epithelialization result, we confirmed the largest collagen deposition on the hypoxic hUC-MSC CM group 14 d post-skin puncture. Correspondingly, Padeta *et al*. [34] reported MSC-CM accelerated collagen deposition in wounded rats compared with the control group. In addition, Li *et al*. [44] found a pronounced effect of MSC CM on keratinocyte proliferation and migration in a high-glucose microenvironment.

## Conclusion

Hypoxia was identified as a better condition to enhance VEGF, bFGF, and pro-collagen 1 secretion. We proved the ability of the hypoxic hUC-MSC CM to facilitate fibroblast cell growth and collagen synthesis through the *in vitro* study. CM from hypoxic hUC-MSC possessed positive and better wound healing effects than antibiotic application on the wound. In addition, wound healing mediated by the hypoxic hUC-MSC CM was based on re-epithelialization and collagen formation evidence in the *in vivo* study. Our findings collectively confirmed that hUC-MSC CM may be used as an alternative treatment to cope with diabetes-related wounds. Our findings might encourage clinical studies that analyze the benefits of hUC-MSC CM for diabetic patients with ulcers.

## Authors’ Contributions

SH, STT, and YK: Conceived, designed, and managed the study. SH and STT: Performed sample collections. YK, CAL, and DF: Prepared human umbilical cord mesenchymal stem cell secretome. SH, JL, and BSM: Did data collection and analysis. SH, EB, BSM, HUB, and STT: Drafted the manuscript. All authors participated in the revision of this manuscript and approved the submission.
